# The impact of traditional cardiovascular risk factor control on 7-year follow-up atherosclerosis progression in systemic lupus erythematosus

**DOI:** 10.1093/rheumatology/kead184

**Published:** 2023-04-22

**Authors:** Nikolaos Papazoglou, Evrydiki Kravvariti, George Konstantonis, Petros P Sfikakis, Maria G Tektonidou

**Affiliations:** First Department of Propaedeutic Internal Medicine, Joint Academic Rheumatology Program, School of Medicine, National and Kapodistrian University of Athens, Athens, Greece; First Department of Propaedeutic Internal Medicine, Joint Academic Rheumatology Program, School of Medicine, National and Kapodistrian University of Athens, Athens, Greece; First Department of Propaedeutic Internal Medicine, Joint Academic Rheumatology Program, School of Medicine, National and Kapodistrian University of Athens, Athens, Greece; First Department of Propaedeutic Internal Medicine, Joint Academic Rheumatology Program, School of Medicine, National and Kapodistrian University of Athens, Athens, Greece; First Department of Propaedeutic Internal Medicine, Joint Academic Rheumatology Program, School of Medicine, National and Kapodistrian University of Athens, Athens, Greece

**Keywords:** SLE, atherosclerotic plaques, cardiovascular risk, target attainment

## Abstract

**Objectives:**

The 2022 EULAR recommendations for cardiovascular risk management in patients with rheumatic disorders, including SLE, call for rigorous management of cardiovascular risk factors (CVRF). The impact of CVRF target attainment on atherosclerotic plaque progression hasn’t been previously evaluated in prospective ultrasound studies.

**Methods:**

A total of 115 patients with SLE and 1:1 age and sex-matched healthy controls who had a baseline carotid and femoral ultrasound examination in our cardiovascular research unit were invited for a 7-year follow-up assessment of new plaque development. We aimed to compare the incidence of plaque progression between SLE patients and controls and reveal the extent to which it is affected by the attainment of European Society of Cardiology (ESC) targets for modifiable CVRFs (blood pressure, smoking status, body weight, lipids and physical activity), and disease-related features (disease duration, disease activity, autoantibodies, treatments).

**Results:**

Eighty-six SLE patients and 42 controls had a 7-year follow-up carotid and femoral plaque examination. New plaque development was observed in 32/86 patients *vs* 8/42 controls (*P* = 0.037). Patients with SLE had a 4-fold higher risk for plaque progression than controls (OR: 4.16, CI: 1.22, 14.19, *P* = 0.023), adjusting for potential confounders. Multivariate regression analyses showed a 50% decrease in plaque progression for every modifiable CVRF fulfilling ESC targets (OR: 0.56, CI: 0.34, 0.93, *P* = 0.026).

**Conclusion:**

Patients with SLE develop a rapid progression of atherosclerotic plaques which may be drastically reduced by CVRF target attainment according to ESC guidelines.

Rheumatology key messages

- SLE carries a 4-fold higher risk for subclinical atherosclerosis progression versus age/sex-matched healthy controls.
- Plaque progression is reduced by 50% for every modifiable CVRF fulfilling the 2016 ESC targets.
- Preventive care in SLE should include rigorous CVRF assessment and management.

## Introduction

SLE is associated with an increased risk of premature atherosclerosis and cardiovascular disease morbidity and mortality [1–5]. Earlier studies, including previous work by our group [6] have shown a 3-fold greater risk for subclinical atherosclerosis in patients with SLE compared with the general population, driven by both traditional and disease-related cardiovascular risk factors (CVRFs) such as the disease duration, disease activity, accrual damage, renal disease, antiphospholipid antibodies and prolonged exposure to corticosteroids [7–9].

In light of the above evidence, the 2022 EULAR recommendations for cardiovascular risk management in rheumatic and musculoskeletal diseases, including SLE and APS, highlighted the importance of thorough assessment and control of modifiable traditional CVRFs in SLE [10], and the role of disease-related CVRF targeting such as disease activity and glucocorticoid exposure. However, the impact of traditional CVRF goal attainment on atherosclerosis progression has not been evaluated in longitudinal studies in SLE. Furthermore, it is not clear whether a long-term lupus low disease activity state (LLDAS) [11] achievement and maintenance may mitigate the risk of accelerated atherosclerosis in patients with SLE.

In the present study, we aimed to assess the risk for the development of new atherosclerotic plaques over a 7-year follow-up period in patients with SLE *vs* healthy controls, traditional and disease-related determinants, and the impact of modifiable CVRF target attainment on carotid and femoral atherosclerotic plaque progression.

## Methods

### Study design and population

**Figure 1.**
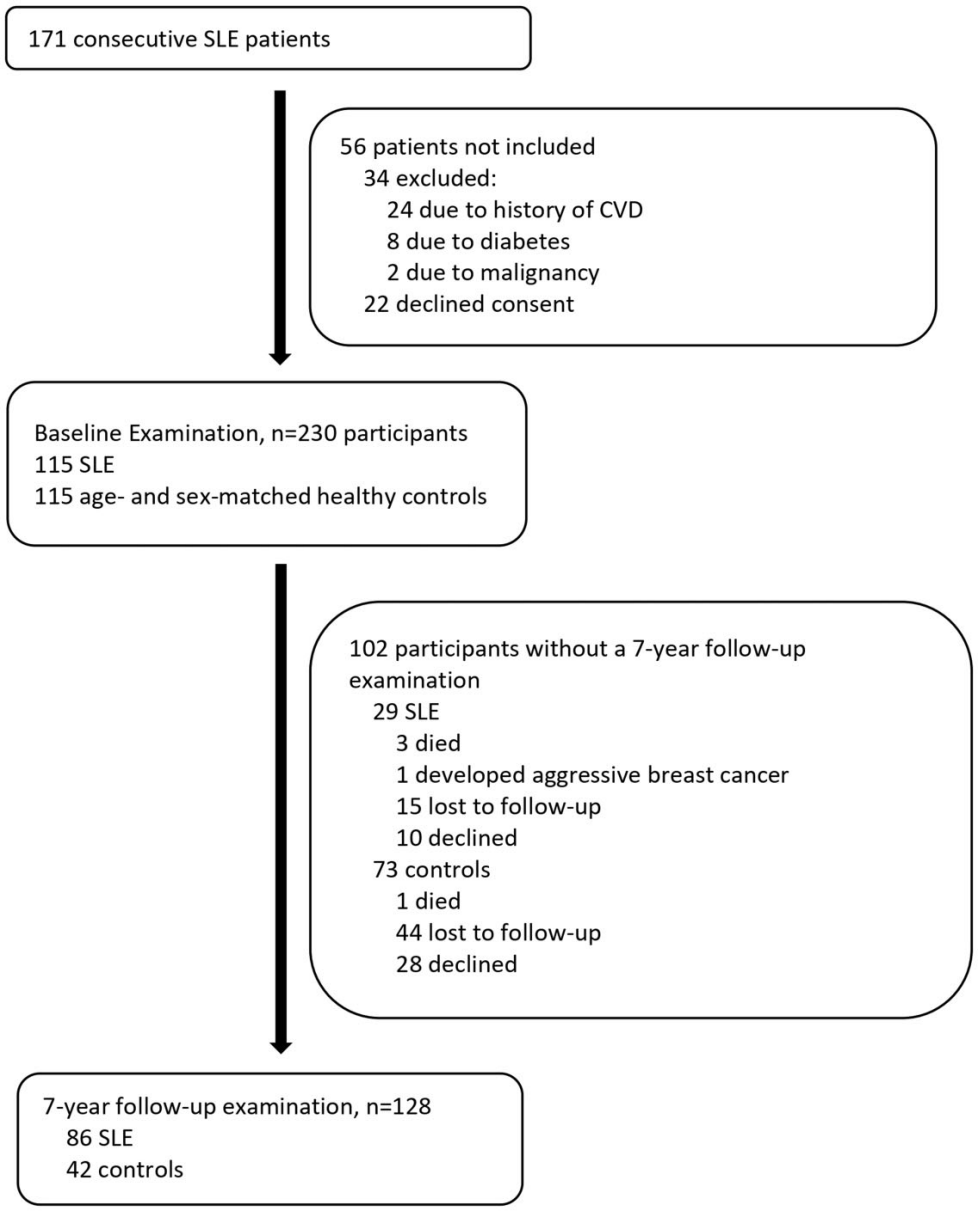
Study flow-chart

This study constitutes an onward vascular ultrasound assessment of participants who were initially evaluated in 2012–2013, when 115 consecutive SLE patients and 115 age/sex matched controls [all without previous atherosclerotic CVD (ASCVD) history] were evaluated by carotid and femoral ultrasound for the presence of subclinical atherosclerotic plaques. All ultrasonographic evaluations and collection of clinical data from control participants were carried out in the Cardiovascular Research (CVR) Laboratory of our department. Since 2010 our CVR laboratory has recruited more than a thousand healthy participants from the local community mainly using flyers and brochures in the university, affiliated hospitals, and neighbouring community centres. Flyers encouraged healthy persons without a need for regular medical follow-up to visit the laboratory for CVRF counselling and screening of subclinical atherosclerosis. Three years after baseline assessment, 101 patients and 85 healthy controls were examined for atherosclerosis progression [12] and in the present study, 86 patients and 42 healthy individuals accepted to participate and had a 7-year follow-up examination (final evaluation held on September 2020). A flow chart is shown in Fig. 1. Between follow-ups, patients had clinical and laboratory evaluations every three to six months. All participants provided written informed consent according to the Declaration of Helsinki and the study was approved by our hospital’s Institutional Review Board (Laiko General Hospital Scientific Council No. 16506).

### Recorded parameters

Demographic parameters including age, sex and ethnicity were initially recorded for all participants. At baseline and at 3-year and 7-year follow-ups, we kept track of data about the following traditional cardiovascular risk factors: (i) systolic and diastolic blood pressure measured after at least 10 min of rest and recorded as the average of three sequential readings taken 1 min apart (Microlife Watch BP Office, Microlife AG, Widnau, Switzerland); (ii) lipid profile data, including total, low- (LDL) and high-density lipoprotein cholesterol (HDL-C) and triglycerides; (iii) smoking status (measured in package-years); (iv) physical activity (measured in min of exercise per week); (v) family history of CVD; (vi) BMI calculated as weight/height^2^; (vii) waist circumference; (viii) the use of antihypertensives, antiplatelets, anticoagulants or statins; and (ix) new documented atherosclerotic CVD (ASCVD), including acute myocardial infarction, acute coronary syndrome, coronary or other arterial revascularization procedures, stroke, peripheral artery disease. New ASCVD events during follow-up were not an exclusion criterion in our study. In order to stratify participants for CVD risk, we used the Systemic Coronary Risk Evaluation (SCORE) prediction, which is a validated instrument recommended by the European Society of Cardiology to estimate the 10-year risk of fatal CVD, taking into consideration age, sex, measurements of systolic blood pressure and fasting total and high-density lipoprotein cholesterol [13].

We further assessed the attainment of therapeutic targets in modifiable CVRFs (namely arterial blood pressure, LDL, HDL, triglyceride levels, smoking status, physical activity level, body mass index, and waist circumference) in both patients and controls, at baseline and follow-up ultrasound assessments. CVRF targets were defined as outlined in the 2016 European Society of Cardiology (ESC) guidelines [14], based on each individual’s cardiovascular disease (CVD) risk classification as low, moderate, high and very high, according to SCORE [13]. Participants with markedly elevated blood pressure (systolic BP≥180 or diastolic BP≥110 mmHg) or lipids (total cholesterol >310 mg/dl) were classified as high-risk, while those with plaque presence on carotid ultrasound were reassigned to the ‘very high-risk’ category. Risk targets in our study followed the 2016 ESC guidelines because the end of the 7-year follow-up assessment for all attendants in the study was before 2021 (last attendant was assessed in September 2020). The 2012 and 2021 ESC guidelines [15, 16] are also mentioned for reasons of comparability (Supplementary Table S1, available at *Rheumatology* online).

### SLE-related factors

Laboratory exams were recorded biannually including complete blood count, ESR, CRP, antinuclear and anti-ds-DNA antibodies, C3 and C4 levels and antiphospholipid antibodies including IgG and IgM anticardiolipin and anti-β2-glycoprotein-1 antibodies, and lupus anticoagulant as defined by the Sydney classification criteria for APS [17]. Medications included corticosteroids (cumulative dose before baseline examination and in the period between baseline and end of follow-up), hydroxychloroquine, immunosuppressants (methotrexate, leflunomide, azathioprine, cyclophosphamide, ciclosporin, mycophenolate mofetil) and biologic agents (rituximab, belimumab).

SLE disease activity was recorded every 6 months at outpatient clinical visits using the SLEDAI 2000 (SLEDAI–2K) [18] including both clinical and laboratory parameters. We further assessed accumulated damage since onset of lupus annually using the SLICC ACR Damage Index (SLICC/ACR DI) [19]. We used the validated definition for lupus low disease activity state (LLDAS): SLEDAI-2K≤4 without major organ involvement, no new disease activity, physician global assessment (0–3)≤1, prednisone≤7.5 mg/day and well-tolerated immunosuppressant dosages [11]. For patients achieving LLDAS, we also calculated LLDAS100, LLDAS75 and LLDAS50, corresponding to maintaining LLDAS for 100%, 75% and 50% of the follow-up time, respectively. We additionally identified patients who remained in clinical remission throughout the 7-year follow-up period (clinical SLEDAI = 0), allowing laboratory activity (persistent anti-ds-DNA and/or low complement), use of hydroxychloroquine, maintenance immunosuppressives and low dose corticosteroids [20].

### Vascular ultrasound and outcome measures

Atherosclerotic plaque was defined as a focal area in the near and far wall of the eight examined carotid and femoral artery sites where intima-media thickness (IMT) was ≥1.5 mm or 50% increased, compared with the adjacent arterial wall IMT, as previously described [6, 12]. Plaque progression was considered present if new plaques were detected at the follow-up ultrasound examination compared with baseline. All baseline and follow-up ultrasound assessments were performed by the same blinded sonographer (G.K.) using a high-resolution B-mode ultrasound device (Vivid 7 Pro, GEHealthcare, Chicago, Illinois, USA) with a 14-MHz multi-frequency linear transducer.

### Statistical analysis

Baseline characteristics representing continuous variables which follow the normal distribution were presented as mean (s.d.), and the *t* test has been applied to control for differences in their mean values between groups. For continuous variables presented as median (Q1–Q3), we have applied the Mann–Whitney *U*. Associations between categorical baseline variables have been tested using Chi^2^. To test multivariate associations after controlling for potential confounders, multiple logistic regression models were applied using plaque progression as the outcome variable. The models included a binary variable representing the participant’s disease setting (0 = controls, 1 = SLE), and a continuous (integer) variable declaring the sum of attained modifiable CVRF targets during follow-up. The sum of targets attained during follow-up represented CVRFs at goal both at 3 years and at 7 years of follow-up including smoking status, physical activity, body weight (BMI and waist circumference), blood pressure and lipids according to 2016 ESC therapeutic targets for traditional CVRF. Baseline characteristics including age, sex, smoking status, BMI, plaque presence and use of hypertension and antiplatelet drugs and statins were also included as categorical co-factors. Given that target attainment in all three lipid parameters has been associated with reduced incidence of cardiovascular disease in other high-risk populations [21], we considered lipids to be within target if LDL, HDL and triglycerides were all on-target. Covariates were pre-selected and force-entered into the models based on their potential confounding impact based on the relevant literature [9, 22, 23].

To explore SLE-related determinants of atherosclerosis progression, we applied multiple logistic regression models in the group of SLE patients only, including the SCORE prediction at baseline, the sum of modifiable CVRF target attainment during follow-up, disease duration and cumulative prednisone during the entire follow-up (continuous variables), use of hydroxychloroquine and LLDAS from baseline to follow-up, antiphospholipid antibody positivity and plaque presence at baseline (categorical variables). Again, covariates were pre-selected based on the relevant literature and force-entered into the models [8, 12, 24–26]. All analyses were performed using the STATA software (version 12.0, College Station, TX, USA).

## Results

### Cohort characteristics

Α total of 230 participants (115 SLE patients and 115 healthy controls, all Caucasians) underwent a carotid and femoral ultrasound at baseline, and after 7 years, 86 eligible SLE patients and 42 healthy controls proceeded to a follow-up ultrasound assessment (Fig. 1). During 7-year follow-up, no participant developed new ASCVD. Characteristics of patients with SLE and controls at baseline and follow-up assessments and differences between groups are shown in Table 1. Compared with controls, SLE patients were more often prescribed antihypertensives and anticoagulants at baseline, statins on follow-up, and antiplatelets both at baseline and on follow-up. Additionally, SLE patients had a higher number of smoking pack-years at both times. No other significant differences were detected between groups. The sum of modifiable CVRF targets attained during follow-up was similar between SLE patients and controls (Table 2).

**Table 1.** Clinical characteristics and plaque progression of patients with SLE and controls

	SLE (*n* = 86)			Controls (*n* = 42)			
	Baseline	3 years FU	7 years FU	Baseline	3 years FU	7 years FU	*P* value*
Age, years	44.4 (11.5)	47.9 (14.2)	51.5 (11.5)	44.9 (14.3)	48.7 (14.2)	51.7 (14.4)	0.892
Female, *n* (%)	78 (90.7)			40 (95.2)			0.369
Follow-up, years [median (IQR)]			7.2 (6.7–7.6)			6.8 (6.3–7.3)	**0.018**
Systolic blood pressure, mmHg [median (IQR)]	116 (109–124)	114 (105–122)	116 (110–131)	119 (109–135)	115 (105–125)	121 (107–137)	0.299
Smoking, pack years [median (IQR)]	5 (0–18)	5.4 (0–18.8)	5.5 (0–21)	0 (0–7)	0 (0–7)	0 (0–7)	**0.004**
Smoking current, *n* (%)	32 (37.2)	30 (34.9)	30 (34.9)	10 (23.8)	9 (21.4)	8 (19.1)	0.130
FH of CAD, *n* (%)	12 (14.0)			3 (7.1)			0.261
Total cholesterol, mg/dl	201 (43)	189 (30)	187 (33)	197 (34)	196 (39)	198 (38)	0.989
LDL, mg/dl	118 (33)	110 (26)	103 (26)	118 (30)	117 (36)	115 (31)	0.663
HDL, mg/dl	62 (22)	60 (16)	62 (17)	61 (14)	60 (12)	61 (11)	0.752
TG, mg/dl	106 (67)	92 (47)	99 (58)	90 (46)	90 (36)	98 (46)	0.177
BMI, Kg/m^2^ [median (IQR)]	24.7 (21.4–28.6)	24.8 (22.7–30.1)	25.5 (22.6–29.9)	25 (21.4–31.2)	26.3 (22.4–30.1)	26.4 (22.3–32.3)	0.539
Exercise, min [median (IQR)]	75 (0–210)	0 (0–180)	112 (0–235)	120 (0–240)	90 (0–180)	0 (0–180)	0.423
Antihypertensives, *n* (%)	31 (36.1)	28 (32.6)	34 (39.5)	7 (16.7)	15 (35.7)	17 (40.5)	**0.024**
Statins, *n* (%)	7 (8.1)	16 (18.6)	21 (24.4)	2 (4.8)	2 (4.8)	3 (7.1)	0.483
Antiplatelet agents, *n* (%)	28 (32.6)	22 (25.6)	24 (27.9)	0 (0.0)	0 (0.0)	2 (4.8)	**<0.001**
Anticoagulants, *n* (%)	16 (18.6)	12 (14.0)	13 (15.1)	2 (4.8)	2 (4.8)	2 (4.8)	**0.034**
SCORE [median (IQR)]	0 (0–1)		1 (0–1)	0 (0–1)		0 (0–2)	0.418
Plaque presence at baseline, *n* (%)	21 (24.2)	33 (38.4)	44 (51.2)	7 (16.7)	9 (21.4)	12 (28.6)	0.319
Plaque progession (new plaques), *n* (%)		16 (18.6)	32 (37.2)		4 (9.5)	8 (19.1)	**0.037**

Values represent mean (s.d.) unless alternately specified.

* *P-*values represent differences between SLE and controls at baseline assessment.

Values in bold indicate differences with statistical significance; *P*-value < 0.05.

FH of CAD: family history of coronary artery disease; FU: follow-up; HDL: high-density lipoprotein; LDL: light-density lipoprotein; SCORE: Systemic Coronary Risk Evaluation prediction of 10-year fatal cardiovascular disease corresponding to 2016 European Society of Cardiology (ESC) guidelines in low-risk countries; TG: triglycerides.

**Table 2.** Cardiovascular risk factor (CVRF) target attainment in patients with SLE and controls

	SLE (*n* = 86)		Controls (*n* = 42)		
	Baseline	During follow-up	Baseline	During follow-up	*P* value^d^
Smoking, *n* (%)	54 (62.8)	53 (61.6)	32 (76.2)	33 (78.6)	0.055
Physical activity, *n* (%)	33 (38.4)	23 (26.7)	19 (45.2)	11 (26.2)	0.947
Body weight (BMI + waist circumference),^a^*n* (%)	32 (37.2)	20 (23.3)	12 (28.6)	6 (14.3)	0.236
Blood pressure, *n* (%)	75 (87.2)	68 (79.1)	32 (76.2)	31 (73.8)	0.504
Lipids (LDL and HDL and Triglycerides),^b^*n* (%)	30 (34.9)	16 (18.6)	20 (47.6)	15 (35.7)	0.034
Sum of CVRF targets attained,^c^ mean (s.d.)	2.6 (1.2)	2.1 (1.1)	2.7 (1.2)	2.3 (1.0)	0.347

a Body weight target was attained if both BMI and waist circumference were on-target.

b Lipids target was attained if LDL, HDL and triglycerides were all on-target.

c Sum of CVRF targets that are attained at baseline and during follow-up representing target attainment both at 3- and 7-year follow-up assessments between: smoking, physical activity, body weight, blood pressure and lipids.

d *P-*values represent differences in CVRF target attainment between SLE and controls during follow-up.

CVRF target attainment according to 2016 ESC guidelines: smoking: no current smoking; physical activity: at least 150 min/week of moderate aerobic physical activity (30 min for 5 days/week) or 75 min/week of vigorous aerobic physical activity (15 min for 5 days/week) or a combination thereof; body weight: BMI 20–25 kg/m^2^ and waist circumference ≥94 cm in men and ≥80 cm in women; blood pressure: systolic blood pressure should be lowered to <140 and diastolic blood pressure to <90 mmHg; lipids: target LDL according to CVD risk category: low-risk to moderate-risk patients: <115 mg/dl, high-risk patients: <100mg/dl or a reduction of at least 50% if the baseline is between 100 and 200 mg/dl, very high-risk patients: <70mg/dl or a reduction of at least 50% if the baseline is between 70 and 135 mg/dl; HDL: no target but >40 in men and >45 in women indicate lower risk; triglycerides: no target but <150mg/dl indicates lower risk and higher levels indicate a need to look for other risk factors.

Detailed disease-related characteristics of patients with SLE can be found in Table 3. Briefly, the mean and median SLEDAI was 2.4 (4.0) and 0 (IQR: 0, 4) at baseline and 1.9 (3.0) and 0 (IQR: 0, 2) at 7-year follow up, while mean and median SLICC was 0.4 (0.5) and 0 (IQR: 0, 1) at baseline and 1.0 (1.0) and 1.0 (IQR: 0, 1) at 7-year follow-up assessment. Thirty-six (41.9%) and 16 patients (18.6%) were in LLDAS and clinical remission throughout their follow-up period, respectively.

**Table 3.** Disease-related characteristics in patients with SLE

	SLE (n = 86)	
	Baseline	7 years FU
Disease duration, mean (s.d.)	8.6 (7.4)	15.7 (7.4)
Antiphospholipid antibody positivity, *n* (%)	32 (37.2)	22 (25.6)
Antiphospholipid syndrome, *n* (%)	18 (20.9)	18 (20.9)
Cumulative prednisone, g [median (iqr)]	5.2 (0.6, 12.5)	13.0 (3.6, 24.9)
Prednisone daily dose during follow-up, mg, [median (iqr)]	NA	1.1 (0, 5.2)
Hydroxychloroquine, *n* (%)	60 (69.8)	74 (86.0)
Hydroxychloroquine use throughout follow-up, *n* (%)	NA	50 (58.1)
Immunosuppressives, *n* (%)	37 (43)	22 (25.6)
SLEDAI, mean (s.d.)	2.4 (4.0)	1.9 (3.0)
SLEDAI [median (IQR)]	0 (0, 4)	0 (0, 2)
SLICC, mean (s.d.)	0.4 (0.5)	1.0 (1.0)
SLICC [median (IQR)]	0 (0, 1)	1 (0, 1)
LLDAS100 during follow-up, *n* (%)	NA	36 (41.9)
LLDAS75 during follow-up, *n* (%)	NA	69 (80.2)
LLDAS50 during follow-up, *n* (%)	NA	81 (94.2)
Clinical remission during follow-up, *n* (%)	NA	16 (18.6)
Renal involvement, *n* (%)	22 (25.6)	22 (25.6)
Central nervous system involvement, *n* (%)	12 (14.0)	12 (14.0)
Pericarditis, *n* (%)	19 (22.1)	19 (22.1)
Alopecia, *n* (%)	11 (12.8)	11 (12.8)
Pleuritis, *n* (%)	9 (10.5)	9 (10.5)
Severe cytopenia, *n* (%)	5 (5.8)	5 (5.8)
Vasculitis, *n* (%)	5 (5.8)	5 (5.8)
Pneumonitis, *n* (%)	1 (1.2)	1 (1.2)

FU: follow-up; NA: non-applicable; LLDAS 100, LLDAS75. LLDAS50: lupus low disease activity state throughout/100%, 75% and 50% of the duration of follow-up, respectively.

Antiphospholipid antibodies were present in 32 (37.2%) patients at baseline and 21 (24.4%) at 7-year follow-up examination and 18 (20.9%) patients fulfilled the Sydney classification criteria for APS. The median daily prednisone dose during the follow-up period was 1.1 mg (IQR: 0, 5.2).

In order to assess any potential differences, we compared baseline cardiovascular and disease activity burden between patients who had a 7-year follow-up assessment and those lost to follow-up (patients who stopped to be followed or declined participation). No differences were found in individual cardiovascular risk factors, such as age, sex, BMI, blood pressure, lipids, baseline and overall SCORE, or use of antihypertensives, anticoagulants and statins, except for current smoking being more prevalent in non-participants (Supplementary Table S2A, available at *Rheumatology* online). In addition, no statistically significant differences were found in baseline SLEDAI, SLICC, major organ involvement, APS co-existence, cumulative prednisolone dose and immunosuppressives use between 7-year participants and patients lost to follow-up (Supplementary Table S2B, available at *Rheumatology* online).

### Plaque progression in SLE *vs* healthy controls

Plaque progression was found in 32 (37.2%) patients with SLE and 8 (19.0%) healthy controls (*P* = 0.037). Among participants with plaque progression, 23 patients (26.7%) and five healthy controls (11.9%) had no plaques detected at baseline assessment (Supplementary Fig. S1, available at *Rheumatology* online). In multivariate regression analysis, patients with SLE exhibited a 4-fold greater risk (OR: 4.16, CI: 1.22, 14.19, *P* = 0.023) for atherosclerosis progression compared with healthy controls after adjustment for age, sex, smoking status, BMI, use of hypertension, lipid-lowering and antiplatelet drugs, plaque presence at baseline and CVRF target attainment (Table 4). The latter had a statistically significant impact on the odds of atherosclerosis progression (OR 0.57 per target reached, CI: 0.32, 0.99, *P* = 0.048), which was similar in both SLE patients and controls (*P* for interaction = 0.210).

**Table 4.** Multivariate logistic regression analysis for the impact of CVRF target attainment on 7-year plaque progression in patients with SLE compared with heathy controls

Plaque progression	OR	*P* value	CI
SLE *vs* healthy controls	4.16	0.023	1.22, 14.19
Sum of CVRF targets attained during follow-up	0.57	0.048	0.32, 0.99
Age	1.12	<0.001	1.06, 1.18
Smoking status	1.23	0.697	0.43, 3.53
Body mass index	0.96	0.555	0.90, 1.06
Antihypertensive drugs	0.50	0.210	0.17, 1.48
Antiplatelet agents	0.89	0.824	0.33, 2.43
Statins	0.71	0.695	0.13, 3.87
Plaque presence at baseline	0.67	0.486	0.22, 2.07

Model adjusted for baseline characteristics including age, sex, smoking status, body mass index, use of antihypertensives, antiplatelet agents, statins and plaque presence.

Sum of CVRF targets attained during follow-up represents CVRF target attainment both at 3-year and 7-year follow-up assessments according to 2016 ESC guidelines between: smoking, physical activity, body weight (BMI and waist circumference), blood pressure and lipids.

### Plaque progression predictors in patients with SLE

Univariate exploratory regression analyses within the SLE cohort are shown in Supplementary Table S3 (available at *Rheumatology* online). Multivariate analysis revealed a 50% reduction in atherosclerotic plaque progression for every additional CVRF meeting ESC targets (OR: 0.56, *P* = 0.026), among smoking, physical activity, body weight, blood pressure and lipids. The only disease-related determinant that was independently associated with plaque progression in SLE patients was disease duration (OR: 1.09, *P* = 0.016, Table 5). Different durations of LLDAS throughout follow-up (LLDAS 50/75/100) and clinical remission were investigated as determinants of atherosclerosis progression and no statistically significant associations arose (Supplementary Tables S3 and S4, available at *Rheumatology* online).

**Table 5.** Multivariate logistic regression analysis models for the impact of CVRF target attainment on plaque progression in patients with SLE

Plaque progression	OR	*P* value	CI
All SLE patients, *n* = 86			
Sum of CVRF targets attained during follow-up (per target)	0.56	0.026	0.34, 0.93
Disease duration	1.09	0.016	1.02, 1.17
SCORE	1.07	0.625	0.81, 1.41
Antiphospholipid antibody positivity	1.29	0.652	0.42, 3.93
Hydroxychloroquine use throughout 7-year follow-up	1.94	0.221	0.67, 5.59
Cumulative prednisone during 7-year follow-up	1.02	0.445	0.96, 1.09
LLDAS100	0.74	0.626	0.22, 2.43
Plaque presence at baseline	0.90	0.349	0.08, 2.44

Sum of CVRF targets attained during follow-up represents target attainment both at 3- and 7-year follow-up assessments according to 2016 ESC guidelines between: smoking, physical activity, body weight (BMI and waist circumference), blood pressure and lipids; SCORE: Systemic Coronary Risk Evaluation prediction of 10-year fatal cardiovascular disease at baseline assessment; LLDAS100: lupus low disease activity state throughout 7-year follow-up.

## Discussion

This is the first study which examined the impact of CVRF target attainment on subclinical atherosclerosis progression in SLE during a long-term follow up. In this 7-year prospective study, we found that patients with SLE exhibit a 4-fold greater risk for carotid and/or femoral plaque progression compared with healthy controls which is halved by 50% for each CVRF target reached by the end of follow-up.

Data from previous, prospective studies with a 3–5 year follow-up documented a 2–3-fold greater risk for subclinical atherosclerosis progression in SLE compared with healthy controls [12, 24]. Our results report a 4-fold higher risk for atherosclerotic plaque progression in a longer, 7-year follow-up, period. The EULAR guidelines for cardiovascular risk management in rheumatic disorders have called for proactive CVD risk assessment and management in this group [10], extrapolating from CVD benefits of this strategy in the general population. Our data confirm that adhering to the ESC guidelines for CVRF management in SLE reduces the incidence of atherosclerotic plaques and may therefore be an efficient cardiovascular prevention strategy. This observation carries high clinical significance, given that it refers to a relatively young population with disproportionally high cardiovascular disease burden.

There are a few prospective studies associating modifiable CVRF accumulation with plaque progression in SLE, namely higher systolic blood pressure [27, 28], dyslipidaemia [27], larger waist circumference [23] and SCORE [12]. However, none of these studies evaluated the impact of the modifiable CVRF goal control, as we did in this study. Some studies have shown that the presence of antiphospholipid antibodies [12, 24], cumulative corticosteroid exposure [12, 29, 30], non-use of hydroxychloroquine [23] and disease duration [12, 28, 31] have been associated with atherosclerosis progression in SLE. Disease duration was the only disease-related characteristic manifesting statistical significance in our multivariate model.

Contradictory to our current results, glucocorticoid use was associated with atherosclerotic plaque progression in previous SLE studies [29, 30] and in the 3-year follow-up study of our SLE cohort [12]. A possible explanation for this could be the relatively low and steadily declining glucocorticoid use in our cohort. Additionally, we failed to detect a protective effect of hydroxychloroquine on subclinical atherosclerosis progression shown in previous studies [32, 33]. This might be explained by the prolonged use of hydroxychloroquine in the majority of our patients or by the less significant effect of hydroxychloroquine on subclinical atherosclerosis markers (e.g. intima media thickness, plaques or coronary calcification) as has been shown in a recent meta-analysis [34] than on cardiovascular events prevention [10]. Congruent with earlier long-term [27] and short-term [29, 31, 35–38] longitudinal studies of subclinical atherosclerosis progression in patients with SLE, no statistically significant association between antiphospholipid antibodies and plaque progression could be established. Finally, we failed to identify a protective effect of maintaining LLDAS on atherosclerosis progression; this may be due to a predominance of patients with low disease activity in our cohort. In addition to the cohort characteristics, our modest sample size may have substantially limited statistical power to reveal an impact of disease-related characteristics.

Our study’s main strengths include the long-term vascular ultrasound follow-up of a well-characterized SLE population examining the impact on atherosclerosis progression of both the disease activity and the CVRF target attainment. One of our limitations is the high loss to follow-up for the healthy control group; however, as it is described in the methods section, they comprised of healthy people without a need for regular medical attention; furthermore, those who declined to participate when they were contacted and interviewed by telephone, did not report an interim development of cardiovascular disease. For these reasons, we believe high rates of loss-to-follow-up in this group of participants represent poor motivation to return or relocation, rather than an informative source of bias. Importantly, this does not interfere with the main finding of our study regarding the impact of CVRF target attainment on atherosclerosis progression in SLE. Because the vast majority of our patients maintained LLDAS for at least 75% of the follow-up time, our results are only applicable to patients with low-disease activity. Due to the cohort composition and modest sample size, our study lacked statistical power to investigate the impact of disease-related features and treatment strategies on atherosclerosis progression. Another limitation is that all participants were Caucasians. There is a pressing need to confirm these findings in larger SLE cohorts of diverse ethnic groups and a broader range of disease activity, which could also assess the potential impact of different treatment strategies on the development of ASCVD in this population.

In conclusion, we have shown that treating CVRFs to target can halt accelerated atherosclerosis in our cohort of patients who maintain low disease activity state and has the potential to alter CVD burden in this population. Further research should be dedicated to the development of valid CVD risk prediction and CVRF management tools specifically designed for this group of patients.

## Supplementary Material

kead184_Supplementary_Data

## Data Availability

The data that support the findings of this study are available by the corresponding author upon reasonable request.
