# Supplementary material for: The impact of traditional cardiovascular risk factor control on 7-year follow-up atherosclerosis progression in systemic lupus erythematosus
Source: Rheumatology (Oxford). 2023 Apr 22;63(1):50–7. doi: 10.1093/rheumatology/kead184 (PMC10765160; doi:10.1093/rheumatology/kead184)
Supplement: kead184_Supplementary_Data [file kead184_supplementary_data.docx]

**Supplementary Material**

The impact of traditional cardiovascular risk factor control on 7-year follow-up atherosclerosis progression in Systemic Lupus Erythematosus

Nikolaos Papazoglou, [Evrydiki Kravvariti](https://pubmed.ncbi.nlm.nih.gov/?term=Kravvariti+E&cauthor_id=30102390), [George Konstantonis](https://pubmed.ncbi.nlm.nih.gov/?term=Konstantonis+G&cauthor_id=30102390), [Petros P Sfikakis](https://pubmed.ncbi.nlm.nih.gov/?term=Sfikakis+PP&cauthor_id=30102390), and [Maria G Tektonidou](https://pubmed.ncbi.nlm.nih.gov/?term=Tektonidou+MG&cauthor_id=30102390)

**Supplementary Table S1.** Therapeutic targets for traditional CVRFs based on 2012, 2016 and 2021 European Society of Cardiology (ESC) Guidelines for CVD prevention in the general population*.*

| **CVD risk factors** | **2012 ESC guidelines for CVD Prevention** | **2016 ESC guidelines for CVD Prevention** | **2021 ESC guidelines for CVD prevention** |
| --- | --- | --- | --- |
| Smoking | no current smoking | no current smoking | no current smoking |
| Physical activity | at least 150 minutes / week  (Healthy adults of all ages should spend 2.5–5 hours a week on physical activity or aerobic exercise training of at least moderate intensity, or 1–2.5 hours a week on vigorous intense exercise) | at least 150 minutes a week of moderate aerobic physical activity (30 minutes for 5 days/week) or 75 minutes / week of vigorous aerobic physical activity (15 minutes for 5 days/week) or a combination thereof | at least 150 - 300 min / week of moderate intensity or 75 - 150 min / week of vigorous intensity aerobic physical activity, or an equivalent combination thereof |
| Body weight | BMI 20–25 kg/m^2^ and Waist circumference ≥94 cm in men and ≥80 cm in women represents the threshold at which no further weight should be gained | BMI 20–25 kg/m^2^ and Waist circumference ≥94 cm in men and ≥80 cm in women: no further weight gain  • Waist circumference ≥102 cm in men and ≥88 cm in women: weight reduction advised | a reduction in weight is recommended for overweight and obese people to improve CVD risk profile (even a moderate weight loss of 5 - 10% from baseline is beneficial)  • Waist circumference ≥94 cm in men and ≥80 cm in women: no further weight gain  • Waist circumference ≥102 cm in men and ≥88 cm in women: weight reduction advised |
| BP | Systolic BP should be lowered to <140 mmHg (and diastolic BP to <90 mmHg) in all hypertensive patients | Systolic BP should be lowered to <140 mmHg and diastolic BP to <90mmHg | The first objective of treatment is to lower BP to <140/90 mmHg in all patients and subsequent BP targets are tailored to age and specific comorbidities:  •In treated patients aged 18-69 years, it is recommended that systolic BP should ultimately be lowered to a target range of 120 - 130 mmHg  •In treated patients aged ≥70 years, it is recommended that systolic BP should generally be targeted to <140 and down to 130 mm Hg if tolerated  •In all treated patients, diastolic BP is recommended to be lowered to <80 mmHg |
| LDL Cholesterol (primary target) | Target LDL according to CVD risk category:   - Low-risk to moderate- risk patients: <115 mg/dl - High-risk patients: <100mg/dl - Very high-risk patients: <70 mg/dl | Target LDL according to CVD risk category:   - Low-risk to moderate -risk patients: <115 mg/dl - High - risk patients: <100mg/dl or a reduction of at least 50% if the baseline is between 100 and 200 mg/dL - Very high-risk patients: <70mg/dl or a reduction of at least 50% if the baseline is between 70 and 135 mg/dL | Target LDL according to age and CVD risk category:   - 40-69 years old: initial goal of LDL <100mg/dl and as second step target LDL<70 mg/dl and ≥50% reduction in high-risk patients and LDL<55 mg/dl and ≥50% reduction in very high-risk patients - ≥70 years old: initial goal of LDL <100 mg/dl. Frailty, polypharmacy and muscle symptoms remain relevant factors to consider in older patients. - Patients with CKD: initial goal of LDL <100 mg/dl and ≥50% reduction and as a second step target LDL<70 mg/dl in high-risk patients and LDL<55 mg/dl in very high-risk patients - Patients with type II DM:  1. Without CVD and/or severe target organ damage: initial goal of LDL <100 mg/dl and as second step target LDL<70 mg/dl and ≥50% reduction 2. With established CVD and/or severe target organ damage: initial goal of LDL <70 mg/dl and as second step target LDL<55mg/dl and ≥50% reduction  - Patients with established CVD: initial goal of LDL <70 mg/dl and ≥50% reduction and as second step target LDL<55 mg/dl |
| HDL-C | Νo target but >40 mg/dl in men and >45 mg/dl in women shows lower risk | No target but >40mg/dL in men and >45 mg/dL in women indicate lower risk | No specific goals for HDL-C levels have been determined in  clinical trials, although low HDL-C is associated with (residual) risk in patients with CVD |
| Triglycerides | Νo target but <150 mg/dl shows lower CVD risk | No target but <150 mg/dL indicates lower risk and higher levels indicate a need to look for other risk factors | No target but <150 mg/dL indicates lower risk and higher levels indicate a need to look for other risk factors |
| HbA1c | - | - | For patients with DM, target HbA1c<7% |

*BMI: body mass index; BP: blood pressure; CKD: chronic kidney disease; CVD: cardiovascular disease; ESC: European Society of Cardiology; HbA1c: haemoglobin A1c; HDL-C: high-density lipoprotein cholesterol; LDL: low-density lipoprotein.*

|  | Participants  (n=86) | Lost to follow-up  (n=25) | p value |
| --- | --- | --- | --- |
| Age, years | 44.4 ± 11.5 | 43.0 ± 13.6 | 0.995 |
| Female, n (%) | 78 (90.7) | 23 (92.0) | 0.841 |
| Systolic blood pressure, mm Hg, [median (IQR)] | 116 (109-124) | 114 (109-124) | 0.988 |
| Smoking current, n (%) | 32 (37.2) | 18 (72.0) | 0.003 |
| Smoking (p/y), [median (IQR)] | 5 (0-18) | 10 (0-28) | 0.302 |
| FH of CAD, % | 12 (14.0) | 3 (12.0) | 0.801 |
| Total cholesterol, mg/dl | 201 ± 43 | 195 ± 50 | 0.595 |
| LDL, mg/dl | 118 ± 33 | 119 ± 46 | 0.955 |
| HDL, mg/dl | 62 ± 22 | 63 ± 24 | 0.684 |
| TG, mg/dl | 106 ± 67 | 95 ± 38 | 0.143 |
| BMI, Kg/m^2^, [median (IQR)] | 24.7 (21.4-28.6) | 24.6 (20.4-30.2) | 0.775 |
| Exercise, min | 75 (0-210) | 0 (0-210) | 0.717 |
| Antihypertensives, n (%) | 31 (36.1) | 9 (36.0) | 0.997 |
| Statins, n (%) | 7 (8.1) | 2 (8.0) | 0.982 |
| Antiplatelet agents, n (%) | 28 (32.6) | 5 (17.2) | 0.223 |
| Anticoagulants, n (%) | 16 (18.6) | 4 (16.0) | 0.766 |
| SCORE, [median (IQR)] | 0.1 (0-1) | 0 (0-1) | 0.510 |
| Plaque presence at baseline, n (%) | 21 (24.2) | 6 (24.0) | 0.966 |

**Supplementary Table S2A**. Baseline characteristics and differences between SLE patients with a 7-year follow-up and those who were lost to follow-up (patients who stopped to be followed or declined participation).

*Values represent mean (S.D.) unless alternately specified. FH of CAD: Family History of Coronary Artery Disease; LDL: Light-Density Lipoprotein; HDL: High-Density Lipoprotein; TG: Triglycerides; BMI: Body Mass Index; SCORE: Systemic Coronary Risk Evaluation prediction of 10-year fatal cardiovascular disease corresponding to 2016 European Society of Cardiology (ESC) guidelines in low-risk countries.*

**Supplementary Table S2B**. Differences in disease-related characteristics in patients with SLE at baseline between 7-year follow-up participants and those who were lost to follow-up (patients who stopped to be followed or declined participation).

|  | Participants  (n=86) | Lost to follow-up  (n=25) | p value |
| --- | --- | --- | --- |
| Disease duration, mean ± SD | 8.6 ± 7.4 | 8.7 ± 7.1 | 0.966 |
| Antiphospholipid antibody positivity, n (%) | 32 (37.2) | 9 (36.0) | 0.866 |
| Antiphospholipid syndrome, n (%) | 18 (20.9) | 4 (16.0) | 0.707 |
| Cumulative prednisone, g [median (IQR)] | 5.2 (0.6-12.5) | 5.9 (0-16.6) | 0.798 |
| Hydroxychloroquine, n (%) | 60 (69.8) | 14 (56.0) | 0.199 |
| Immunosuppressives, n (%) | 37 (43) | 8 (28.0) | 0.176 |
| SLEDAI, mean ± SD | 2.4 ± 4.0 | 2.5 ± 4.0 | 0.912 |
| SLEDAI, [median (IQR)] | 0 (0, 4) | 0 (0, 4) | 0.912 |
| SLICC, mean ± SD | 0.4 ± 0.5 | 0.3 ± 0.5 | 0.219 |
| SLICC, [median (IQR)] | 0 (0, 1) | 0 (0, 1) | 0.219 |
| Renal involvement, n (%) | 22 (25.6) | 5 (20.0) | 0.705 |
| Central nervous system involvement, n (%) | 12 (14.0) | 4 (16.0) | 0.679 |
| Pericarditis, n (%) | 19 (22.1) | 7 (28.0) | 0.404 |
| Alopecia, n (%) | 11 (12.8) | 3 (12.0) | 0.974 |
| Pleuritis, n (%) | 9 (10.5) | 4 (16.0) | 0.363 |
| Severe cytopenia, n (%) | 5 (5.8) | 0 (0.0) | 0.236 |
| Vasculitis, n (%) | 5 (5.8) | 0 (0.0) | 0.236 |
| Pneumonitis, n (%) | 1 (1.2) | 1 (4.0) | 0.312 |

*SLEDAI: Systemic Lupus Erythematosus Disease Activity Index; SLICC: Systemic Lupus International Collaborating Clinics*

**Supplementary Figure S1**. 7-year plaque progression in patients with SLE and healthy controls

| **Plaque progression** | **OR** | **P value** | **CI** |
| --- | --- | --- | --- |
|  |  |  |  |
| Sum of CVRF targets attained during follow-up | 0.60 | 0.021 | 0.39 – 0.93 |
| Disease duration | 1.09 | 0.007 | 1.02 – 1.16 |
| SCORE | 1.10 | 0.484 | 0.84 – 1.43 |
| Antiphospholipid antibody positivity | 1.56 | 0.335 | 0.63 – 3.82 |
| Hydroxychloroquine use throughout 7-year follow-up | 1.33 | 0.529 | 0.55 – 3.26 |
| Cumulative prednisone during 7-year follow-up | 1.01 | 0.583 | 0.97 – 1.06 |
| LLDAS100 during follow-up | 0.64 | 0.333 | 0.26 – 1.58 |
| LLDAS75 during follow-up | 1.33 | 0.631 | 0.42 – 4.26 |
| LLDAS50 during follow-up | 0.56 | 0.569 | 0.07 – 4.17 |
| Clinical Remission during follow-up | 0.44 | 0.083 | 0.18 – 1.11 |

**Supplementary Table S3**. Univariate logistic regression analysis for plaque progression in patients with SLE during the 7-year follow-up.

*Sum of CVRF targets attained during follow-up: CVRF target attainment both at 3- and 7-year follow-up assessments between: smoking, physical activity, body weight (body mass index and waist circumference), blood pressure and lipids according to 2016 ESC guidelines; SCORE: Systemic Coronary Risk Evaluation at baseline assessment; LLDAS100, LLDAS75, LLDAS50: Lupus Low Disease Activity State throughout 100%, 75 and 50% of the duration of follow-up, respectively.*

**Supplementary Table S4**. Multivariate logistic regression analysis models for the impact of CVRF target attainment on plaque progression in patients with SLE (sensitivity analysis including clinical remission instead of LLDAS)

|  | **OR** | **P value** | **CI** |
| --- | --- | --- | --- |
| **All SLE patients, n=86** |  | | |
| Sum of CVRF targets attained during follow-up (per target) | 0.54 | 0.010 | 0.33 – 0.86 |
| Disease duration | 1.09 | 0.019 | 1.01 – 1.17 |
| SCORE | 1.06 | 0.693 | 0.80 – 1.40 |
| Antiphospholipid antibody positivity | 1.13 | 0.832 | 0.37 – 3.50 |
| Hydroxychloroquine use throughout 7-year follow-up | 1.95 | 0.221 | 0.67 – 5.66 |
| Cumulative prednisone during 7-year follow-up | 1.00 | 0.936 | 0.95 – 1.06 |
| Clinical Remission | 0.37 | 0.118 | 0.11 – 1.28 |
| Plaque presence at baseline assessment | 0.90 | 0.876 | 0.25 – 3.27 |

*LLDAS: Lupus Low Disease Activity State; Sum of CVRF targets attained during follow-up: CVRF target attainment both at 3- and 7-year follow-up assessments between: smoking, physical activity, body weight (body mass index and waist circumference), blood pressure and lipids according to 2016 ESC guidelines; SCORE: Systemic Coronary Risk Evaluation at baseline assessment.*
